# One-Electron Oxidation Potentials and Hole Delocalization in Heterogeneous Single-Stranded DNA

**DOI:** 10.1021/acs.biochem.3c00324

**Published:** 2023-11-03

**Authors:** Jesús Lucia-Tamudo, Manuel Alcamí, Sergio Díaz-Tendero, Juan J. Nogueira

**Affiliations:** † Department of Chemistry, Universidad Autónoma de Madrid, Madrid 28049, Spain; ‡ Institute for Advanced Research in Chemical Sciences (IAdChem), Universidad Autónoma de Madrid, Madrid 28049, Spain; § Condensed Matter Physics Center (IFIMAC), Universidad Autónoma de Madrid, Madrid 28049, Spain

## Abstract

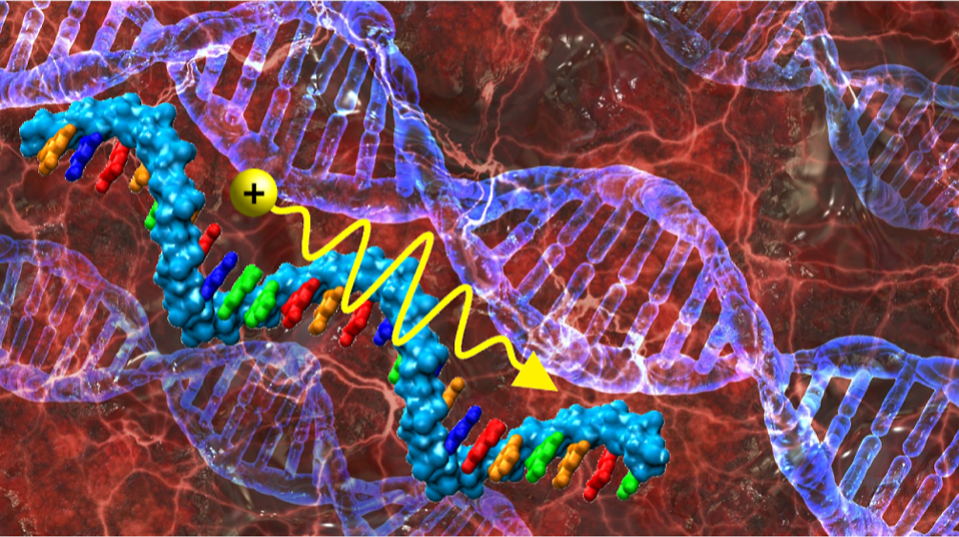

The study of DNA processes is essential to understand not only its intrinsic biological functions but also its role in many innovative applications. The use of DNA as a nanowire or electrochemical biosensor leads to the need for a deep investigation of the charge transfer process along the strand as well as of the redox properties. In this contribution, the one-electron oxidation potential and the charge delocalization of the hole formed after oxidation are computationally investigated for different heterogeneous single-stranded DNA strands. We have established a two-step protocol: (i) molecular dynamics simulations in the frame of quantum mechanics/molecular mechanics (QM/MM) were performed to sample the conformational space; (ii) energetic properties were then obtained within a QM1/QM2/continuum approach in combination with the Marcus theory over an ensemble of selected geometries. The results reveal that the one-electron oxidation potential in the heterogeneous strands can be seen as a linear combination of that property within the homogeneous strands. In addition, the hole delocalization between different nucleobases is, in general, small, supporting the conclusion of a hopping mechanism for charge transport along the strands. However, charge delocalization becomes more important, and so does the tunneling mechanism contribution, when the reducing power of the nucleobases forming the strand is similar. Moreover, charge delocalization is slightly enhanced when there is a correlation between pairs of some of the interbase coordinates of the strand: twist/shift, twist/slide, shift/slide, and rise/tilt. However, the internal structure of the strand is not the predominant factor for hole delocalization but the specific sequence of nucleotides that compose the strand.

## 1 Introduction

The information regarding the characteristics of every living organism is stored in nucleic acid molecules RNA or DNA. Specifically, DNA is the macromolecule responsible for this function in eukaryotic organisms. Over the decades, humans have attempted to determine which regions of the chromosomes encode each gene and the sequence that determines its expression. However, the functionalities of DNA have been expanded during the last few decades.^1^ Among these innovative applications, its use in DNA computation,^2,3^ DNA-templated synthesis for new materials,^4^ molecular detection,^5−15^ and as a nanowire^16,17^ can be highlighted. In the case of the latter two applications, the modes of operation are similar. These systems consist of an ensemble of single-stranded DNA (ss-DNA) or double-stranded DNA (ds-DNA) anchored to a metallic surface, in the case of an electrochemical biosensor, or two electrodes, in the case of a nanowire. In the first stage, there is a charge transfer between a component of the system, such as an analyte or an electrode, and the DNA strand. Generally, nucleobases are the primary moieties responsible for the charge transfer process in aqueous phase.^18,19^ Thus, a comprehensive understanding of the redox properties of nucleobases is crucial for gaining insights into this phenomenon. Specifically, nucleobases are more prone to oxidation than reduction. This implies that a positive charge is generated within the nucleobase. As a result, obtaining an accurate value of the one-electron oxidation potential is essential, and numerous studies have been conducted to elucidate them.^20−31^ This property can be understood as the reduction potential of an oxidation process. In this context, the relative order of the reducer character for free nucleobases in water is well-known: G > A > T ∼ C > U. In fact, in previous works, we found a clear relationship between the number of atoms of a nucleobase in which the positive charge is delocalized and the relative order of the reducer character within nucleobases.^32^

In the second stage, after the generation of the positive charge, hole transport occurs along the DNA strand. Since nucleobases hold the hole in water, these moieties are also responsible for the transport of the positive charge. In this context, two main mechanisms have been proposed: tunneling and hopping.^33−36^ On the one hand, tunneling advocates for transport based on the hole delocalization along several nucleobases until it reaches the receiving component.^34^ This mechanism shows a dependency on the distance between nucleobases: two nucleobases must be close enough to enable hole delocalization through π-stacking interactions. On the other hand, hopping arises as an alternative to the tunneling model to explain the long-range transport of the charge in DNA. It is a multistep process that states that the charge is localized in just one nucleobase and moves through consecutive jumps from one nucleobase to another with similar redox properties. In fact, nucleobases with identical one-electron oxidation potentials can transfer the charge to one another, even if other nucleobases are interspersed between them. In contrast to the tunneling model, the dependency on distance becomes less relevant for the hopping model. Thus, by consecutive hopping processes, the charge can be transferred over long distances. It is said that guanine and, to a lesser extent, adenine are hopping stones for hole transfer, while thymine and cytosine are hopping stones for electron transfer. However, both processes, hole and electron transfer, do not have the same probability of occurring because of the different rates that they present. While hole transfer can take place from a nanosecond to a microsecond time scale, electron transfer takes from minutes to weeks. In a previous work, we determined that the hopping model is likely predominant over the tunneling model for the case of homogeneous ss-DNA.^32^ This is supported by the evidence that charge delocalization becomes less relevant due to the stabilization produced by solvent effects; the charge tends to be mainly held in a single nucleobase, especially when the solvent has a high polar character and the nucleobase has a high reducer character.^37,38^

**Figure 1:**
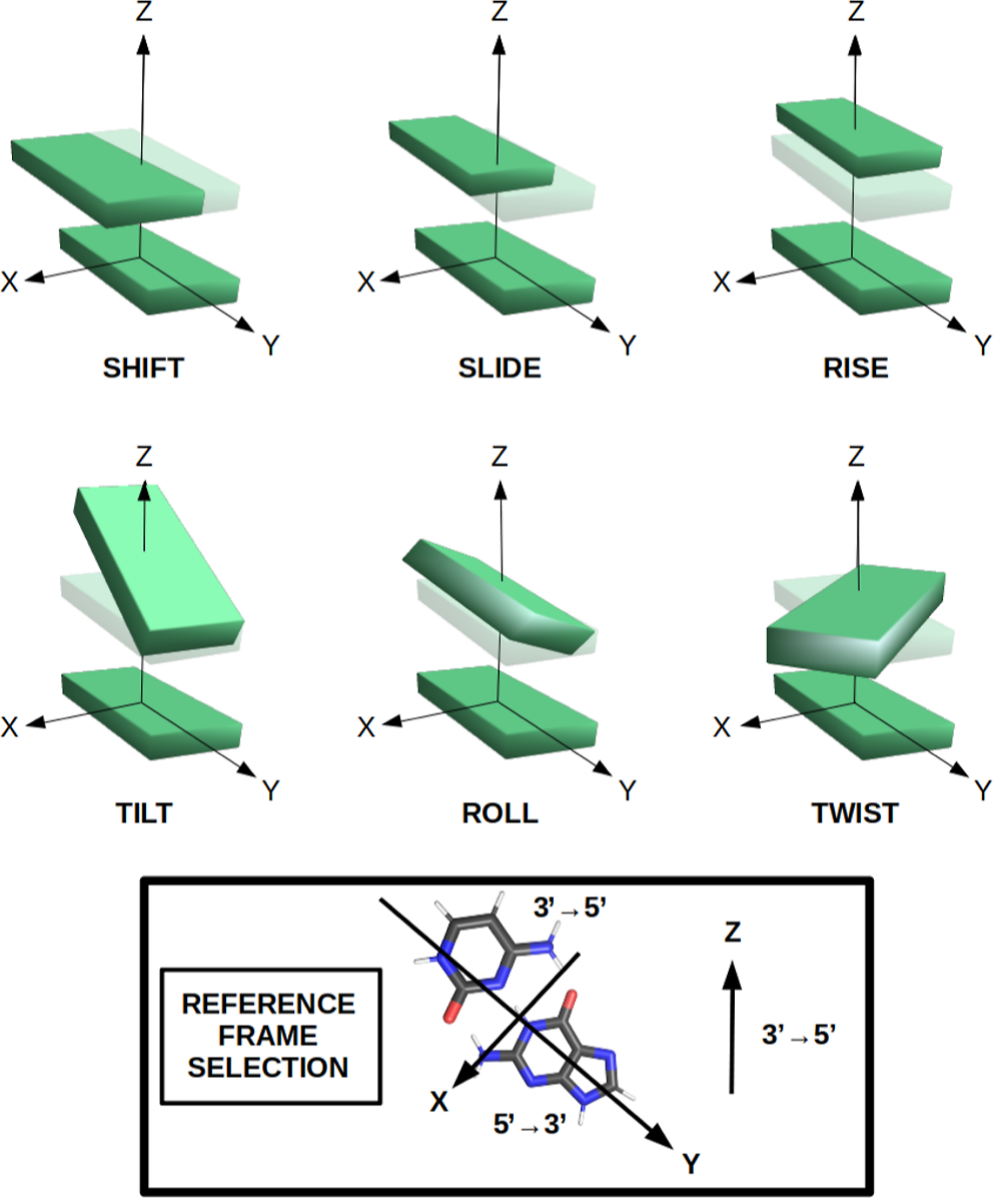
Graphical description of the interbase pair parameters. The reference frame selection is also displayed to fix the convention of the parameters. The systems under study follow the 5′ → 3′ direction.

The need to understand the causes of this transport puts the focus on the delocalization feature. Since DNA is a considerably large biomolecule, analyzing all degrees of freedom simultaneously to find a relationship between the conformation of the strand and the delocalization of the hole along it becomes unfeasible. Fortunately, the complex structure of DNA has led to the convention of some parameters that allow for the analysis and comparison of different conformations of a specific strand.^39−41^ In general terms, the conformation of ds-DNA strands can be described by six sets of parameters: helical axis, base pair-axis, intrabase pair, interbase pair, backbone parameters, and groove parameters. The helical axis is described by its general stretching and torsion along the strand. The base pair-axis set accounts for the deformation of the axis between two adjacent base pairs. The interbase pair set describes the arrangement of two adjacent base pairs (see Figure 1), and the intrabase pair set provides insights into the arrangement of the two nucleobases that compose the base pair. The puckering of the sugar of each nucleotide gives information about the conformation of the backbone of the strand. Finally, the groove parameters provide information about the major and minor grooves in a ds-DNA strand. However, when this analysis is applied to an ss-DNA strand, the sets that account for the intrabase pair and groove parameters cannot be defined.

In this work, we have determined the one-electron oxidation potential of heterogeneous ss-DNA strands and compared them with those obtained for homogeneous ss-DNA in a previous study.^32^ Additionally, we have computed the delocalization of the hole along these ss-DNA strands and found a relationship between this phenomenon and the interbase pair structural parameters, as shown in Figure 1.

## 2 Methods and Computational Details

The computation of the one-electron oxidation potential and the delocalization properties of the strands were conducted using the same procedure employed in a previous study on homogeneous ss-DNA.^32^ After the setup of the different solvated ss-DNA strands, a conformational sampling was carried out using classical and quantum mechanics/molecular mechanics (QM/MM) molecular dynamics (MD) simulations. Subsequently, the properties were computed for an ensemble of geometries selected from the QM/MM trajectories through electronic structure calculations. These calculations were performed by using a QM1/QM2/continuum approach in combination with the Marcus theory and electron population analysis. In the following, the specific details of all of these steps are deeply discussed.

**Figure 2:**
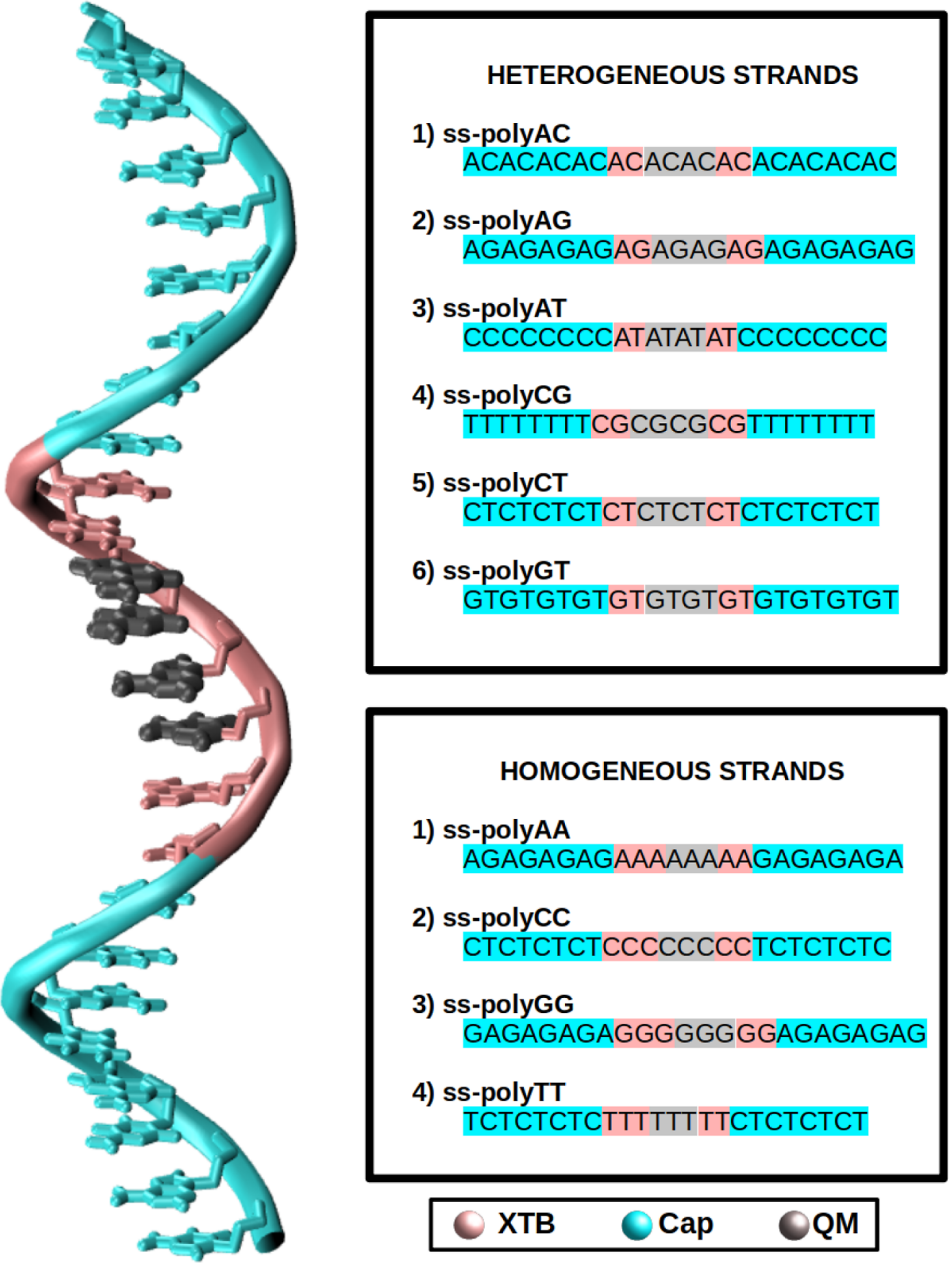
Graphical view of the general form of the systems under study in this work. The full sequence for each system is also displayed for ss-polyX and ss-polyXY. The color of the strand represents the layer to which these nucleobases and nucleotides belong. Cyan refers to the nucleotides that form protective caps of the strand. Gray refers to the nucleobases that are included in the QM1 region. Pink is associated with the backbone of the QM1 region and nucleotides that belong to the XTB (QM2) layer.

The nucleic acid builder (NAB) application provided by the AmberTools 22 package^42−44^ was used to model the initial geometries of the heterogeneous ss-DNA strands. Each of the six strands investigated was composed of 24 nucleotides, as shown in Figure 2. Specifically, the eight nucleobases of the center of the strands correspond to tetramers of all possible combinations of nucleobase pairs: (AC)_4_, (AG)_4_, (AT)_4_, (CG)_4_, (CT)_4_, and (GT)_4_. To prevent self-hybridization, especially in ss-polyCG and ss-polyAT, a limiting cap of 8 nucleotides was added to each edge of the strands. The ss-DNA strands were solvated in a truncated octahedron box with a buffer of 12 Å, and the tleap program implemented in AmberTools 22 was used for this purpose. The ff90bsc0 force field,^45,46^ along with the dihedral correction addressed in bsc1,^47^ was selected to describe ss-DNA, while the TIP3P force field^48^ was used for describing water molecule interactions. To counteract the negative charge of the strands, 22 sodium cations were added using the parameters developed by Joung and Cheatham.^49^ In order to compare the behavior of ss-polyXY, homogeneous ss-polyX systems, taken from ref (32), were also included and analyzed in this study. Specifically, the system sizes are the following ones: 63,462 atoms in ss-polyA, 63,935 atoms in ss-polyC, 63,728 atoms in ss-polyG, 63,372 atoms in ss-polyT, 63,697 atoms in ss-polyAC, 63,613 atoms in ss-polyAG, 63,740 atoms in ss-polyAT, 63,975 atoms in ss-polyCG, 63,722 atoms in ss-polyCT, and 63,703 atoms in ss-polyGT.

The configurational space was explored through classical MD simulations^50−52^ using the CUDA version of the pmemd program implemented in the AMBER 20 package.^42−44^ The simulations for the homogeneous ss-polyX systems were obtained from a previous study,^32^ while the same procedure was applied for the heterogeneous ss-polyXY. The simulations began with a 10,000-step minimization, where the first 5000 steps were computed using the steepest-descent algorithm,^53^ followed by another 5000 steps using the Newton–Raphson algorithm.^54^ A constant volume (*NVT*) progressive heating to 300 K was then run for 500 ps, and a thermostat was applied according to the Langevin model with a collision frequency of 2 ps^–1^ to regulate the temperature. After that, an additional 500 ps simulation was conducted at a constant temperature of 300 K (*NVT* ensemble). In the following stage, a 1 ns simulation was run in the *NPT* ensemble to equilibrate the volume of the system and achieve the correct density. Finally, a production simulation of 200 ns was conducted in the *NPT* ensemble, and 200 equidistantly separated snapshots were selected. The Berendsen barostat with isotropic position scaling and a pressure relaxation time of 2 ps were used for all simulations carried out within the *NPT* ensemble to maintain the pressure constant at 1 bar. The particle-mesh Ewald method with a grid spacing of 1.0 Å was employed to compute the electrostatic interactions during the full protocol, and a 10 Å cutoff was chosen for the nonbonded interactions. The SHAKE algorithm^55−57^ restrained the bonds involving hydrogen atoms, and a time step of 2 fs was used during the heating, equilibration, and production stages.

In order to compute the one-electron oxidation potential, *E*_red_, we used the Marcus theory formulation^58−63^ that states that
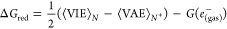
1where VIE (vertical ionization energy) is the energy required to remove an electron from a neutral species, VAE (vertical attachment energy) is the energy released upon adding an electron to a cationic species, and *G*(*e*_(gas)_^–^) = −0.867 kcal/mol is a correction for the free energy of the electron in the gas phase, calculated using the Fermi–Dirac statistics.^64−66^

Notice that both VIE and VAE are required to compute the one-electron oxidation potential within Marcus theory. While VIE can be easily obtained from the snapshots of the aforementioned classical dynamics, calculating VAE requires a conformational sampling of the phase space of the cationic strand. Unfortunately, force field parameters are not available for the cation, making calculation of VAE a challenge. To overcome this limitation, a set of 200 QM/MM MD simulations for each strand was conducted using as initial conditions an ensemble of 200 snapshots selected from each of the classical MD simulations. Thus, the combination of classical and QM/MM MD guarantees a statistically accurate thermal distribution of the solvent molecules, while the relevant region of the DNA is described quantum mechanically.

The QM/MM dynamics trajectories were carried out for both neutral and cationic phase spaces to ensure consistency. Specifically, additional 100-step QM/MM MD simulations were run in the *NPT* ensemble for each of the selected frames using the ORCA^67^/AMBER interface. The computational details of these dynamics are the same as those of classical MD simulations. The interaction between the QM and MM layers was described by an electrostatic embedding approach, where the MM charges were taken from the ff90bsc0^45,46^ (along with the corrections from bsc1)^47^ and the TIP3P^48^ force fields for DNA and water, respectively. The QM region, comprising four adjacent nucleobases, was computed using the CAM-B3LYP functional^68^ and the 6-311G(d)^69,70^ basis set. It is important to mention that the ensemble of 200 geometries selected from each classical trajectory follows a Boltzmann distribution, making it an appropriate representative ensemble of each system. As a result, these QM/MM MD simulations are solely intended to refine the structure of the QM region of the strands, while the solvent was already equilibrated along the classical simulation. In this way, 200 quantum mechanically described geometries are obtained from each simulation, which will be used for the analysis in this study. The number of considered geometries for each system proved to be more than enough to obtain converged values of the properties under study, as can be reflected in Figure S1 in the Supporting Information.

Finally, the last geometry obtained from each QM/MM MD simulation was used to calculate the VIEs and VAEs for each heterogeneous ss-DNA strand. The calculations were carried out using a QM1/QM2/continuum scheme, where the QM1/QM2 interaction was described by an electrostatic embedding and the QM/continuum interaction was modeled by a polarizable embedding. The VIEs and VAEs were computed for the QM1 region, where four nucleobases were described with the CAM-B3LYP/6-311G(d) level of theory. After removing the limiting caps, the remaining four nucleobases in the QM2 region were described by tight-binding DFT (DFTB) using the GFN2-xTB scheme.^71^ The atomic charges from the QM1 and QM2 regions were computed at the corresponding level of theory of the layer. Solvent effects were taken into account using the ALPB continuum solvation model,^72^ which is suitable for DFTB. Thus, the QM region is polarized by the solvent described by this continuum solvation model. All calculations were performed using the ORCA 5.0.3 package.^67^

The free energy computed by eq 1 can be related to the one-electron oxidation potential through the following equation
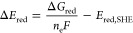
2where *F* is the Faraday constant, *n*_e_ is the number of exchanged electrons (one in this case), and *E*_red,SHE_ is the reduction potential of a reference electrode, which in this case is the standard hydrogen electrode (SHE). The considered value of *E*_red,SHE_ was 4.281 V, used in previous works.^73−77^ This value also accounts for the correction of the free energy of the electron in the gas phase. As a result, this contribution must also be added in eq 1.

The delocalization of the hole was analyzed by calculating the charge difference of each nucleobase in the QM1 region between the cationic and neutral species for each of the geometries of the ensembles (200 geometries for each strand). The Löwdin charges^78^ were used for charge calculations, and the analysis was conducted using custom scripts. The intermolecular delocalization number, denoted as *n*, was defined as the number of nucleobases, among which the positive charge is distributed after ionization. To determine *n*, the four nucleobases considered in the QM1 region were first ordered in terms of increasing positive charge difference Δ*q*_*i*_, and then an empirical equation, whose details can be found in a previous work, was applied^32^
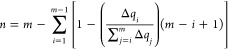
3Note that the term  represents the contribution to the delocalization of each nucleobase. Additionally, the term (*m* – *i* + 1) indicates the number of nucleobases over which this delocalization contribution is taken into account. To sum up, the total number of nucleobases where the charge is delocalized, *n*, is obtained as the number of considered nucleobases *m* minus the noncontribution to delocalization of each one.

In this context, Pipek and Mezey reported another way to quantify the delocalization of a positive charge among a system.^79^ In this case, they obtained a delocalization index using the atomic Mulliken population of the set of orbitals in each atom. In other words, to compare our empirical eq 3 with the equation by Pipek and Mezey, we have adapted the latter one by considering the charge difference of each nucleobase of the QM region leading to
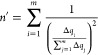
4

**Figure 3:**
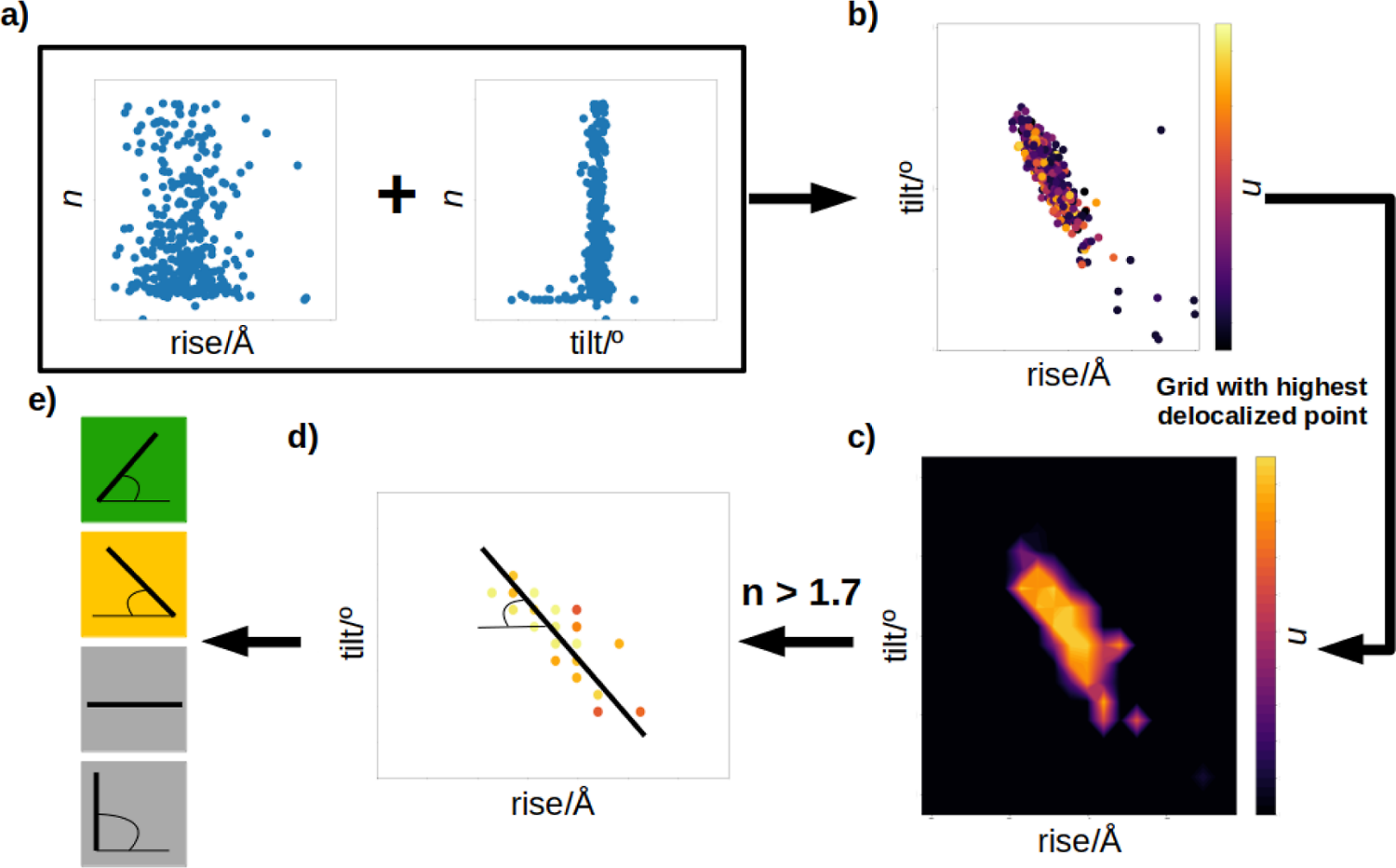
Graphical explanation of the discrimination of the data to obtain correlations between pairs of parameters. (a) Relative *n* plotted against one parameter. (b) Relative *n* plotted against two parameters. (c) Grid that contains the maximum delocalization among the sampled conformational space. (d) Points from the grid whose relative *n* is larger than 1.7 and linear regression over those points. (e) Classification of the correlation between a pair of parameters.

Once the delocalization index was determined, structural analysis was performed to investigate the relationship between the conformation of the strands and the delocalization of the hole. The following procedure was applied for both homogeneous and heterogeneous ss-DNA (as shown in Figure 3).

First, the structural parameters of the selected geometries were obtained using the CURVES+ package.^39−41^ The conformational space explored, thanks to the MD simulations, can be found in Figure S2 in the Supporting Information. In this study, only the interbase pair set of parameters were analyzed (see Figure 1). The convergence of the value of each of these parameters is displayed in Figure S3a in the Supporting Information, as well as the distributions of the sampled parameters for each strand (see Figures S4–S9). For each pair of nucleobases within the QM1 region, the relative intermolecular delocalization number *n* was computed and plotted against each of the interbase pair parameters (see Figures 3a and S10 in the Supporting Information). From that analysis, no significant conclusions could be extracted, concluding that the dependence of delocalization on each parameter might not be independent for each of them and, in contrast, could follow complex patterns that combine the effects of several of them simultaneously. As a result, a three-dimensional representation of *n* as a function of each pair of structural parameters was plotted as a contour map to see possible correlations between the structural parameters and large values of charge delocalization (see Figure 3b). The limits of those conformational spaces (for each structural parameter) were set to ±2.5σ, where σ represents the standard deviation of each parameter along the geometrical ensemble. Therefore, each of the axes of Figure 3b goes from −2.5σ to 2.5σ and the center of each axis is located at the mean value of the corresponding geometrical parameter. Although these limits are chosen in an arbitrary manner, they cover the whole conformational space where delocalization is allowed.

In order to identify the region of the conformational space with the largest positive charge delocalization, the previous contour maps were simplified in the following way. A 20 × 20 grid was applied to each resulting conformational space. For each element of the grid, the point with the largest delocalization number was selected and plotted, while the rest were discarded (Figure 3c). Thus, only those points with large hole delocalization were chosen to analyze the role of the structural parameters. Moreover, further discrimination was conducted by only considering elements of the grid with an *n* higher than 1.7, while the rest were discarded (see Figure 3d). Finally, a linear regression was performed with these remaining points to observe the correlation of each pair of parameters with respect to the delocalization. Each pair of interbase pair parameters was classified into four groups in terms of the resulting slope of the regression once normalized (see Figure 3e). The normalization was performed by considering that a slope equal to 1 is obtained when the straight line goes in the direction from the point (−2.5σ, −2.5σ) to the point (2.5σ, 2.5σ) and equal to −1 when it goes from (−2.5σ, 2.5σ) to (2.5σ, −2.5σ). Thus, if the slope was near 1 and −1, then the pair was considered positively and negatively correlated, respectively, when charge delocalization is important. On the contrary, if the slope was closer to 0 or to ∞, then one of the parameters did not show a correlation with the other one when delocalization was large. An identical analysis was conducted for those situations in which the delocalization number gives values lower than 1.1, which means in cases where delocalization can be neglected (see Figure S11I in the Supporting Information). In addition, a similar procedure was performed using all of the points regardless of the value of *n* to complement the study (see Figure S11II). In this case, the set of points obtained in Figure 3b was adjusted to a straight line in terms of the pair of parameters.

## 3 Results

### 3.1 One-Electron Oxidation Potential and Charge Delocalization

The reducing power of a specific species can be measured in terms of its reduction potential. When the target reaction involves an oxidation process in which one electron is involved, the reduction potential is commonly referred to as the one-electron oxidation potential, as stated previously. Thus, determining this property can establish a hierarchy in terms of the reducing power of a set of molecules. In this work, the one-electron oxidation potential was computed for different sequences of nucleotides within an ss-DNA strand. In a previous study, this property was determined for sequences with identical nucleotides labeled as ss-polyX (X = A, C, G, T).^32^ Here, the target strands are composed of two different nucleotides interspersed with one another, and therefore, these systems will be labeled as ss-polyXY (X, Y = A, C, G, T, and X ≠ Y). Our previous results indicate that the computation of the one-electron oxidation potential of large systems involving nucleobases can be accurately conducted by combining Marcus theory with MD simulations and QM/continuum models.^31^ Therefore, the one-electron oxidation potential was determined for ss-polyXY following this methodology, including four nucleobases in the QM1 region as explained above.

**Figure 4:**
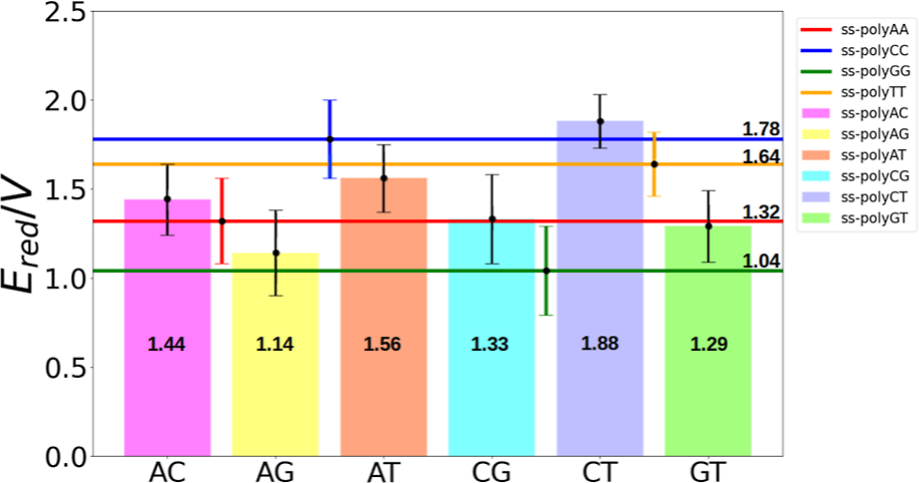
Computed one-electron oxidation potentials for ss-polyX (solid lines) and ss-polyXY (bars) in the aqueous phase. The *E*_red_ values for ss-polyX are taken from ref (32). These one-electron oxidation potentials are obtained from the QM region of each considered strand (three nucleobases in ss-polyX and four nucleobases in ss-polyXY).

The results show that changes in the potential depend on the number of nucleotides of each type present in the strand, as seen in Figure 4. Specifically, the *E*_red_ of ss-polyXY takes a value between the two limiting situations of ss-polyX and ss-polyY. The only exception to this trend is in the case of ss-polyCT, for which the *E*_red_ is slightly higher than those of both pure ss-DNA strands. However, the value lies within the standard deviation of the calculations. These results reflect that *E*_red_ can be seen as a linear combination of the potentials of pure single strands formed by the nucleobases present in the heterogeneous one. This is consistent with the experimental results obtained by Capobianco et al. and rationalized by Nardi et al.^80,81^ They observed that the presence of guanines adjacent to another guanine in an ss-DNA strand (the ss-polyG case in Figure 4) reduced the one-electron oxidation potential with respect to the case in which a guanine was surrounded by thymine moieties (ss-polyGT case in Figure 4). They predicted that the decrease of *E*_red_ was ∼0.1 V per each adjacent guanine, which is in good agreement with our results: the oxidation potential goes from 1.29 for ss-polyGT, where there are no adjacent guanines, to 1.04 for ss-polyG, where there are 2 consecutive guanines adjacent to the target guanine. In the present study, we have extended the analysis to all the possible combinations XGX where X = A, C, T and we observed that this trend is always accomplished independently on the nucleobase that surrounds the guanine moiety; that is, the oxidation potential for polyG is always lower than that of polyGX.

In this sense, a strand that contains purines, which are highly reducing nucleobases, will be more reducing than another strand containing pyrimidine nucleobases. The reducer character of the nucleobases and the reducing character difference between them will be intimately related to the charge delocalization, as will be discussed below. However, from this analysis, it is not possible to determine if there is a predominance of one of the nucleobases with respect to the other in the linear combination. Since we cannot provide an exact value of the property but a range of values, we cannot state which ss-polyX system dominates the resulting potential of ss-polyXY. It is also interesting to highlight that, in previous works, it has been evidenced that the one-electron oxidation potential of the nucleobases decreases significantly when these molecules belong to a DNA strand with respect to the case in which they are isolated.^32^ Specifically, the *E*_red_ of each isolated nucleobase in water was found to be 1.58 V for adenine, 2.02 V for cytosine, 1.27 V for guanine, and 2.06 V for thymine. In the case of ss-polyXY, this trend is also observed. The one-electron oxidation potential of the strands investigated here is typically lower than that of the isolated nucleobase with the most reducing power. This is in good agreement with the trend predicted by Nardi et al., where they rationalized this increase in the reducer potential when the nucleobase forms part of a strand.^81^ There are only two cases in which this statement is not accomplished: ss-polyCG and ss-polyGT. This is due to the high potential of cytosine and thymine, which means that the resulting potential of the strand increases considerably.

**Figure 5:**
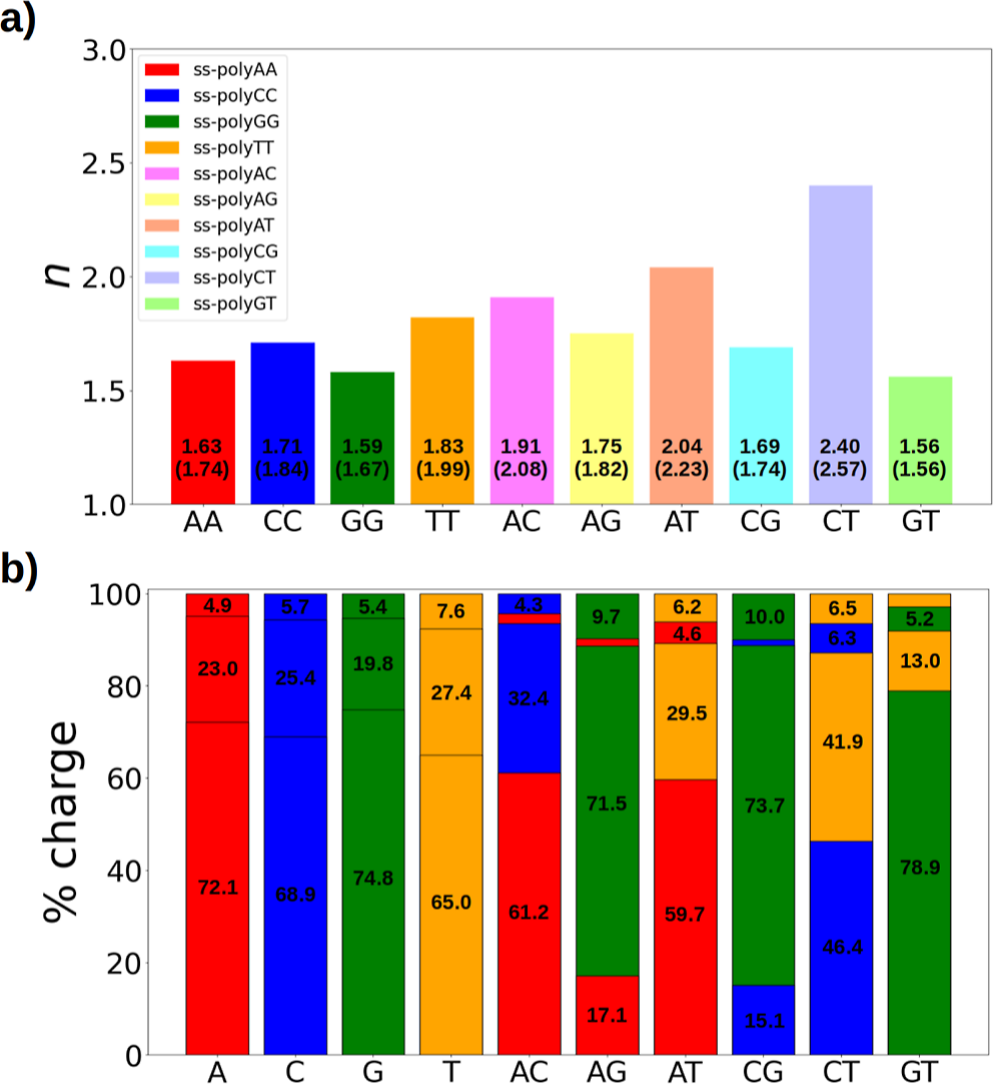
Delocalization of the hole along the strands considered. (a) Intermolecular delocalization number for each ss-polyX and ss-polyXY. *n* (*n*′) values are displayed within the corresponding bars. (b) Percentage of positive charge held by each nucleobase of the strand. The colors of (b) represent the type of nucleobase: A in red, C in blue, G in green, and T in orange. The results from ss-polyX were taken from ref (32). All of these results are the average value of the property obtained from the QM region (three nucleobases in ss-polyX and four nucleobases in ss-polyXY) of the ensemble of considered geometries.

After oxidation, the positive charge (hole) can be transferred along the strand by different mechanisms. On the one hand, the charge can be delocalized among several nucleobases, evolving in space with time according to the tunneling mechanism. On the other hand, the transport can be conducted through sequential jumps from one nucleobase to another by the hopping mechanism, in which the charge is essentially localized in just one nucleobase at a time. In order to get insights into the dominant mechanism in ss-DNA strands, the delocalization of the hole along the different ss-polyXY strands considered has been assessed using eq 3. For comparison, the delocalization numbers have also been calculated using eq 4, and the results are shown in Figure 5a. Although the values for *n*′ are slightly higher than those for *n*, the relative order for the different ss-DNA strands remains invariant. Therefore, in the following, only the *n* values are discussed for simplicity. Figure 5 displays the values of *n* for the different strands (panel a) and the amount of charged particles hosted by each of the nucleobases of the strands included in the QM region (panel b). The charge delocalization numbers for ss-polyX strands are taken from a previous work^32^ and are also shown for comparison.

It has previously been shown that there exists a competition in homogeneous ss-polyY strands between intrabase and interbase delocalization of the hole.^32^ Greater intramolecular delocalization to accommodate the positive charge was obtained for purines, which have a larger π system than for pyrimidines. Contrarily, when considering homogeneous ss-DNA systems of cytosines and thymines, where the π system is more spatially constrained, intramolecular delocalization is reduced compared to purines, and thus, the interbase delocalization of the hole becomes more important. Based on the results shown in Figure 5, a similar situation is found in heterogeneous ss-polyXY, although now the delocalization depends on two factors: (i) the oxidation potential of the most reducing nucleobase of the strand, and (ii) the difference in the reducing character of the two nucleobases. The importance of each of these factors depends on the composition of the strand.

When guanine is part of the strand, the properties are clearly dominated by those of guanine. As shown in Figure 5a, the delocalization numbers of ss-polyAG, ss-polyCG, and ss-polyGT are small and similar to that of ss-polyGG since the hole is preferably located on only one guanine, as can be seen in Figure 5b. Specifically, around 70–80% of the positive charge is hosted by one of the guanine nucleobases when guanine is part of the strand. In addition, the charge is more delocalized in ss-polyAG than in ss-polyCG or ss-polyGT because the reducing characteristics of adenine and guanine are more similar than those for guanine and thymine/cytosine. Thus, the positive charge is shared between both nucleobases, although with significant dominance of guanine, increasing the delocalization number. Contrarily, cytosine and thymine are not able to attract the positive hole because their reducing power is much smaller than that of guanine.

When guanine is not present in the strand, the competition between nucleobases for hosting the charge is greater. In this way, the delocalization number for ss-polyAC and ss-polyAT is larger than that when guanine is present and it is also larger than that of ss-polyAA. This is also reflected in the fact that now only 65% of the charge is located on one of the adenine moieties in ss-polyAC and ss-polyAT, while around 30% of the charge is located on thymine or cytosine. Finally, when two pyrimidines are combined (ss-polyCT), the largest intermolecular delocalization (2.4) is obtained compared to the other binary combinations and with ss-polyCC and ss-polyTT (see Figure 5a). Both nucleobases have small π systems, and thus, the intramolecular delocalization is small and intermolecular delocalization is preferred. In addition, both nucleobases have similar one-electron oxidation potentials, and thus, none of the nucleobases has a preference to host the positive charge of the hole in ss-polyCT. As represented in Figure 5b, the two cytosine molecules included in the QM region accommodate 46.4 and 6.3% of the charge, while a similar situation is found for the two thymines. This means that the charge delocalization is evenly shared between cytosine and thymine, increasing further the delocalization number.

Our analysis of the charge delocalization supports a hopping mechanism with some contribution of tunneling for the transport of the hole along the ss-DNA strands. While the positive charge is predominantly localized on one nucleobase in most of the cases, there is always a certain degree of delocalization of the charge toward the nucleobase adjacent to the predominant one. In addition, the extent of the tunneling character increases as the potential between the two nucleobases present in the strand is more similar, for example, in ss-polyCT and ss-polyAT.

### 3.2 Relation between Structure and Charge Delocalization

**Figure 6:**
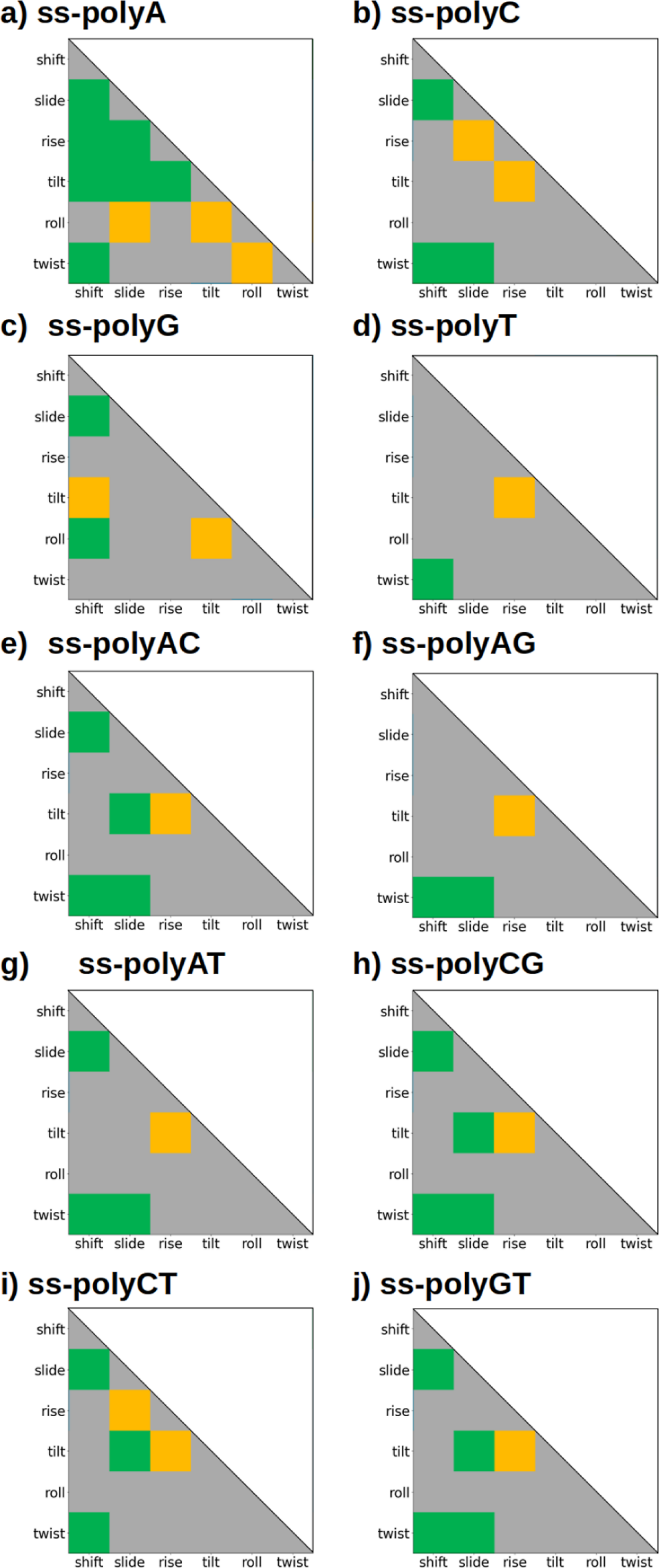
Colored matrix representation of the existing correlations between interbase pair parameters in ss-DNA. Since the matrices are symmetric, only the lower triangle is displayed. Color code: green accounts for positive correlation, orange represents negative correlations, and gray refers to nonexisting correlation.

In order to obtain the previously discussed results of the one-electron oxidation potential and delocalization, classical MD simulations were performed, followed by QM/MM MD simulations, with the aim of exploring the conformational space of the different systems. The sampled conformational space of each system is analyzed here in terms of the shift, slide, and rise distance parameters and tilt, roll, and twist angle parameters. These parameters are related to the charge delocalization following the analysis explained above and schematically displayed in Figure 3. The results of this analysis are presented in Figure 6 in the form of color matrices, representing the type of correlation that exists between pairs of structural parameters that lead to the largest delocalization number of the positive charge between pairs of nucleobases. The gray color indicates that there is no correlation between the pair of parameters. Green color accounts for positive correlation; that is, the highest charge delocalization is obtained when both structural parameters decrease or increase at the same time. Negative correlation is represented in yellow, meaning that the highest delocalization is achieved when one of the parameters increases while the other decreases. As aforementioned, a similar study can be found in the Supporting Information (see Figure S11I) with the lowest delocalization in order to compare both regions. Finally, it is worth mentioning that these matrices are symmetric and the diagonal does not have any physical meaning. As a result, only the lower triangle of each color matrix is presented.

As can be seen in Figure 6, a certain degree of similarity can be observed in the correlation matrices of all of the strands studied except the ss-polyA one. In general terms, there exist three commonly found positive correlations: one between twist and shift, which is present in 9 out of 10 strands, another between shift and slide, present in 8 out of 10 strands and, to a lesser extent, another between twist and slide, found in 6 out of 10 systems. The reason behind the positive correlation between these pairs of parameters is to enhance the overlap between the aromatic rings of the consecutive nucleobases to strengthen the interaction between these monomers. For example, the natural torsion of ss-DNA and ds-DNA strands is the origin of a significant twist value, a fact that weakens the interactions between nucleobases. The enhancement of the π-stacking interactions, leading to a large charge delocalization, can be achieved by the displacement of one of the interacting nucleobases along the *X*-axis increasing, thus, the shift distance or along the *Y*-axis, i.e., increasing the slide distance. Therefore, the increase of the twist angle requires the increase of the shift value to favor the delocalization of the hole. Another less common positive correlation that was found along the dynamics of some ss-DNA strands is slide/tilt (in 5 out of 10 strands). Although these relationships are also present in some of the systems for the case with low delocalization (see Figure S11I in the Supporting Information), their frequency is lower, giving evidence that these relationships lead to situations with enhanced hole delocalization.

Figure 6 also shows that there is a recurrent negative correlation between the rise distance and the tilt angle. Specifically, for 8 out of 10 strands, when the rise distance decreases, the tilt angle increases. In this case, this negative correlation is likely aimed at avoiding strong repulsive interactions between the neighboring nucleobases. The repulsion originated by small rise distances between consecutive nucleobases can be alleviated when one of the nucleobases is tilted, inducing an increase in the separation of the aromatic clouds. As in the positive correlations, this relationship is also present in the low-delocalization regime. Nevertheless, the number of appearances is lower, and it can be stated that this correlation is also stronger in the high-delocalization regime. In conclusion, when the configurational space that accounts for strong positive charge delocalization is analyzed, shift, slide, and twist correlate positively to increase the attractive interactions, while the rise/tilt pair correlates negatively to decrease the repulsive interactions. These correlations appear, in general, along the whole range of values of *n* (see Figure S11II). This means that these correlations are intrinsic properties of the structure of ss-DNA. However, it is more frequent to find these associations in the high-delocalization regime than in the low-delocalization regime. Therefore, one can conclude that these structural correlations between pairs of parameters favor the enhancement of the hole delocalization along an ss-DNA strand. However, this effect does not seem to be the predominant factor that controls the extent of delocalization. Instead, delocalization is more dominated by the sequence of nucleobases disposed along the strand and the individual capability of each of them to delocalize the charge.

## 4 Conclusions

In this computational study, the one-electron oxidation potential and the degree of delocalization of the positive hole formed after oxidation in heterogeneous ss-polyXY have been investigated. The results have been compared with other analogues obtained from homogeneous ss-polyX. In addition, a structural analysis has been carried out to study the effect of the correlation between some structural parameters on the delocalization of the hole along the strand and shed light on the importance of the two hole transport mechanisms in DNA, namely, tunneling and hopping.

The results show that the one-electron oxidation potential of ss-polyXY takes a value between the two limiting situations ss-polyX and ss-polyY, and can be seen as a linear property in terms of the composition of the system in the case of ss-DNA. Thus, a strand containing purine nucleobases will be more reducer than one formed by pyrimidine nucleobases. When the degree of delocalization of the hole among adjacent nucleobases in ss-DNA is analyzed, the results reveal that the delocalization number depends on the oxidation potential of the most reducing nucleobase and on the reducing character difference between the two nucleobases present in the strand. When guanine is one of the components of the system, its properties are dominated by those of guanine. It has been computed that around 80% of the hole charge is located on just one of the guanine moieties. Contrarily, when guanine is not forming part of the strand, the delocalization number increases due to an increase in the competition between the different nucleobases to host the charge. Such a competition is more important when the nucleobases of the strand have similar reducing power, for example, as in ss-polyCT. Therefore, our computational analysis supports the idea that the hole is transported along ss-DNA strands mostly by a hopping mechanism with some tunneling contribution. Such a tunneling component will be more relevant when guanine is not present.

The structural analysis of the dynamics shows that large charge delocalization is achieved when some of the structural parameters of the strand are correlated. Although these relationships are also observed when no delocalization is present, the correlation seems to become stronger when the hole can be delocalized along different nucleobases. On one side, the positive correlation between twist, shift, and slide enhances the attractive interactions between nucleobases. On the other side, the negative correlation between rise and tilt reduces the repulsion between nucleobases. These correlations likely lead to a larger overlap between the aromatic clouds of the nucleobases, a fact that induces a slightly larger charge delocalization. However, charge delocalization is not dominated by these structural correlations but by the nucleobase sequence present along the strand.

## Supporting Information Available

Convergence of electronic properties with respect to the number of geometries considered, conformational space explored along the MD simulations, convergence of the interbase pair parameters among the conformational sampling, representative conformations observed throughout the MD simulations, distribution of the amount of hole delocalization with respect to the interbase pair parameters, and relation between structural parameters in absence of delocalization (PDF)
