# Supplementary material for: One-Electron Oxidation Potentials and Hole Delocalization in Heterogeneous Single-Stranded DNA
Source: Biochemistry. 2023 Nov 3;62(22):3312–22. doi: 10.1021/acs.biochem.3c00324 (PMC10666269; doi:10.1021/acs.biochem.3c00324)
Supplement: Supplementary file 1 — bi3c00324_si_001.pdf [file bi3c00324_si_001.pdf]

Supporting Information for 'One-Electron Oxidation Potentials and Hole Delocalization in Heterogeneous Single-Stranded DNA'

Jesús Lucia-Tamudo,[†] Manuel Alcamí,[†,‡] Sergio Díaz-Tendero,[*,†,‡,¶] and Juan J. Nogueira[*,†,‡]

†*Department of Chemistry, Universidad Autónoma de Madrid, 28049, Madrid, Spain*

‡*Institute for Advanced Research in Chemical Sciences (IAdChem), Universidad Autónoma de Madrid, 28049 Madrid, Spain*

¶*Condensed Matter Physics Center (IFIMAC), Universidad Autónoma de Madrid, 28049 Madrid, Spain*

E-mail: sergio.diaztendero@uam.es; juan.nogueira@uam.es

1 Convergence of Electronic Properties

In Figure S1, the cumulative value of VIE and the number of delocalization n_{VIE} for neutral trajectories are displayed, as well as the cumulative value of VAE and the number of delocalization n_{VAE} for cationic trajectories. In a few cases, some of the cationic or neutral single-points did not converge and, as a result, the associated curves do not reach the 200 frames. Based on the results, it can be concluded that VIE (VAE) reaches a constant value for all strands after considering approximately 50 geometries. Convergence of the delocalization number values requires a larger number of geometries, although it is observed that after 100 conformations, the value of this property remains stable. Consequently, it can be

stated that the use of 200 geometries for calculating the average value of all these properties is more than enough. In fact, for those systems for which the cumulative curve does not reach the 200 snapshots, the number of the discarded geometries is very small and does not affect the accuracy of the results.

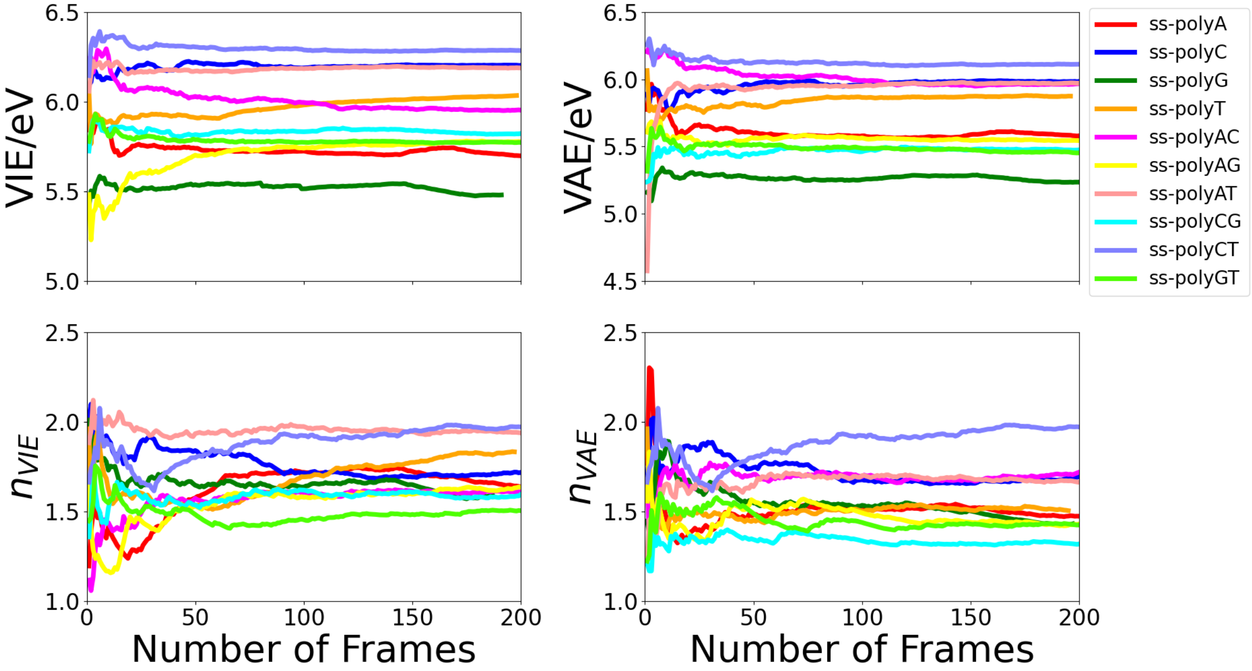

Figure S1: Cumulative values of the VIE, VAE and delocalization number for neutral (n_{VIE}) and cationic trajectories (n_{VAE}) in terms of the number of frames.

2 Convergence of the Conformational Sampling

Figure S2 shows the conformation sampling carried out by the MD simulations represented by the range ($-\sigma+\langle A\rangle$,$+\sigma+\langle A\rangle$) where σ is the standard deviation and $\langle A\rangle$ is the mean value of the parameter obtained from the 200 considered geometries. The range of the conformational exploration in ss-DNA is larger than in ds-DNA due to the higher flexibility of the first ones. Still, the highest delocalization is typically found for all the strands at similar values of each parameter. As a result, the deviation of the point with highest delocalization of the hole with respect to the average value is small in the vast majority of the cases. This gives

evidence that there should be some relationships between the inter-base pair parameters and the amount of delocalization common to all the strands. This motivation led to the structural analysis carried out in the main text of this work.

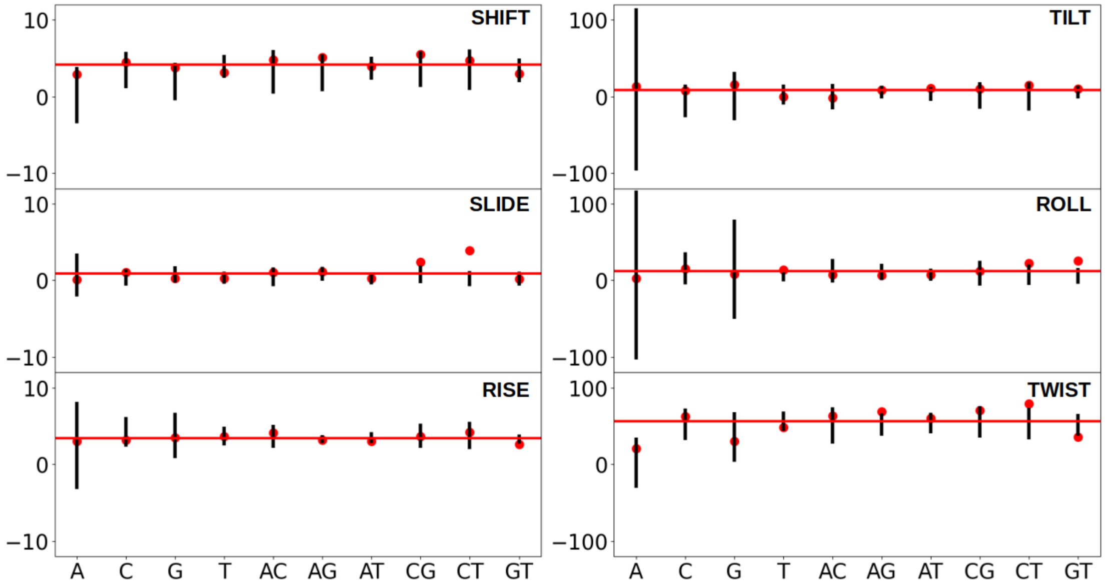

Figure S2: Conformational sampling of the inter-base pair parameters (shift, slide, rise, tilt, roll and twist). Black vertical lines represent the range $(-\sigma,+\sigma)$, where σ is the standard deviation of the parameter, centered with respect to the mean value of the parameter along the trajectory. Red points indicate the value of the parameter for which the higher delocalization was found. Finally, red lines accounts for the mean value of the parameter with the higher delocalization.

Additionally, a convergence analysis of the inter-base pair parameters was conducted, which is displayed in Figure S3a. Note that absolute values have been taken into account to avoid artifacts raised from the cancellation of positive and negative values. For the vast majority of the cases, each parameter reached a constant value after 100 snapshots. Moreover, these constant values were found to be similar for almost all the ss-DNA systems. However, there are two main exceptions: ss-polyA and, to a lesser extent, ss-polyG. Usually, the nucleobases are parallel one another as displayed in Figure S3c. For the case of ss-polyA the cumulative value of the inter-base pair parameters differs considerable from the rest of the strands. This could mean that these systems have reached another stable conformation

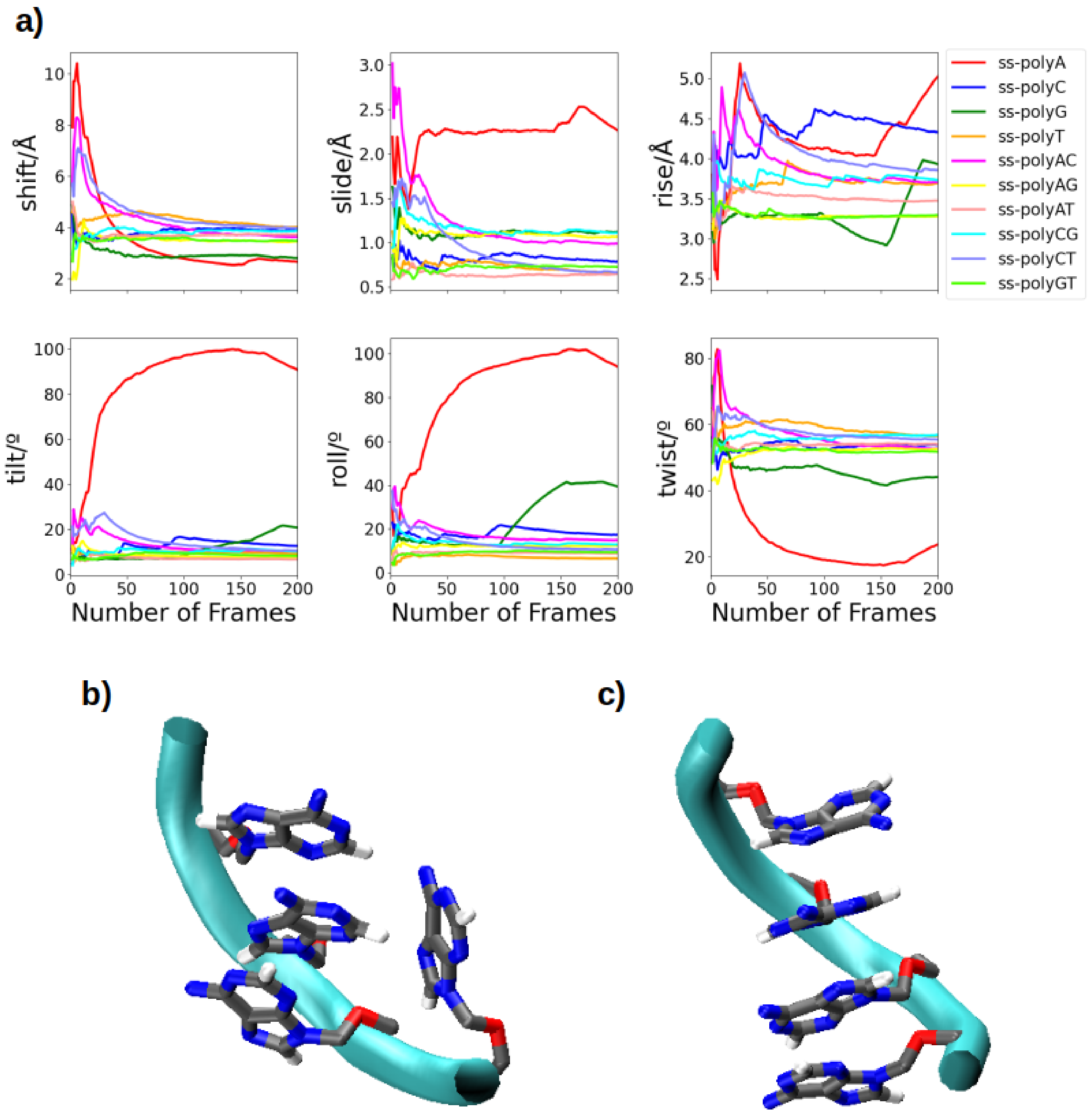

Figure S3: a)Cumulative absolute values of the inter-base pair parameters (shift, slide, rise, tilt, roll and twist) in terms of the number of frames. The panels below show representative frames of the b) t-shape, c) parallel conformation of nucleobases in ss-polyA.

different from the rest of the systems where the nucleobases are disposed in a t-shape relative position (see Figure S3b). This makes that the tilt and the roll angles increase considerably while the twist angle decreases. As a result, one of the nucleobases is placed along the z-axis, which makes the rise distance increase significantly. In addition, the slide displacement is also

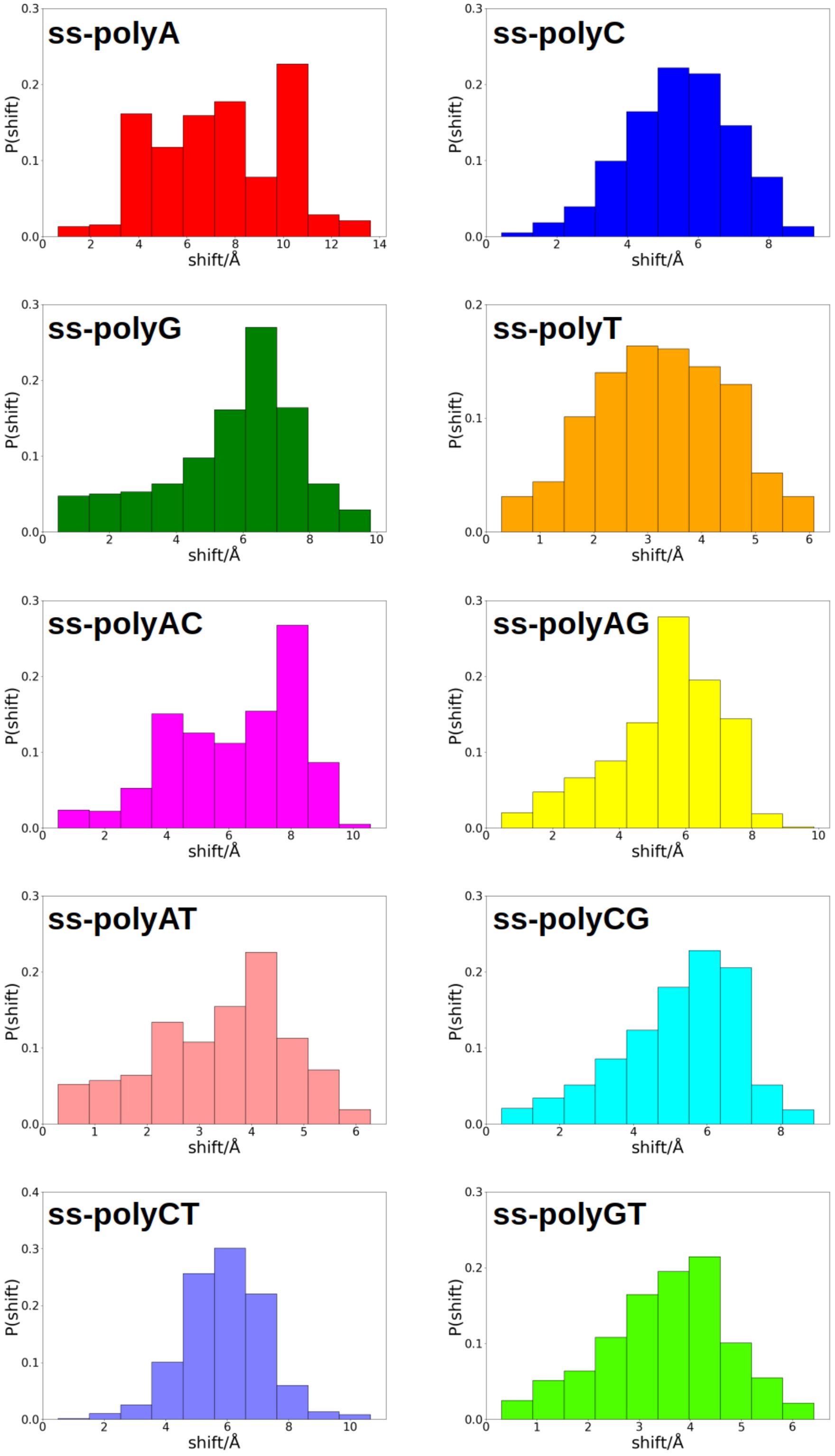

Figure S4: Distributions of the sampled shift parameter along the simulations of each ss-DNA strand.

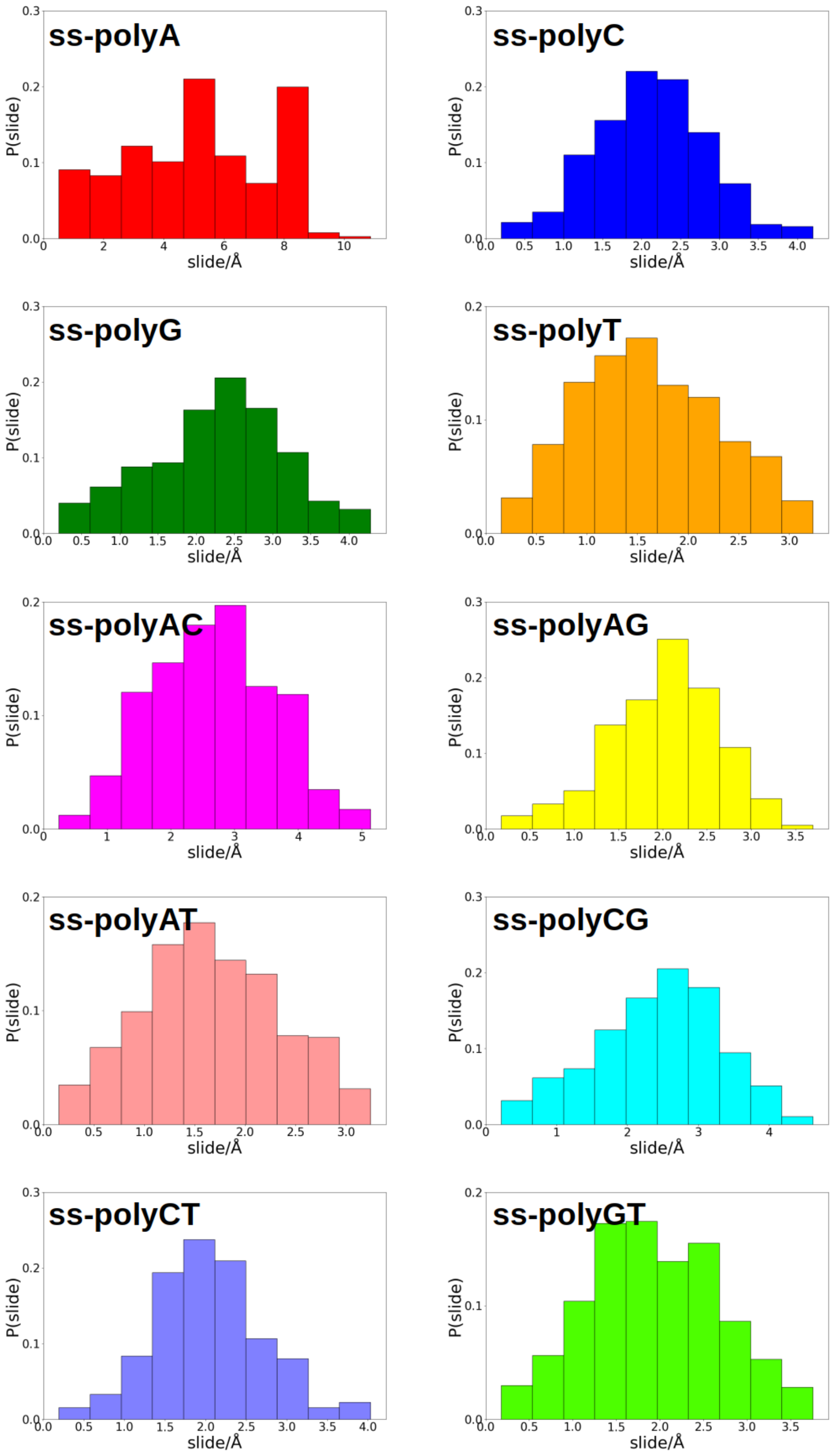

Figure S5: Distributions of the sampled slide parameter along the simulations of each ss-DNA strand.

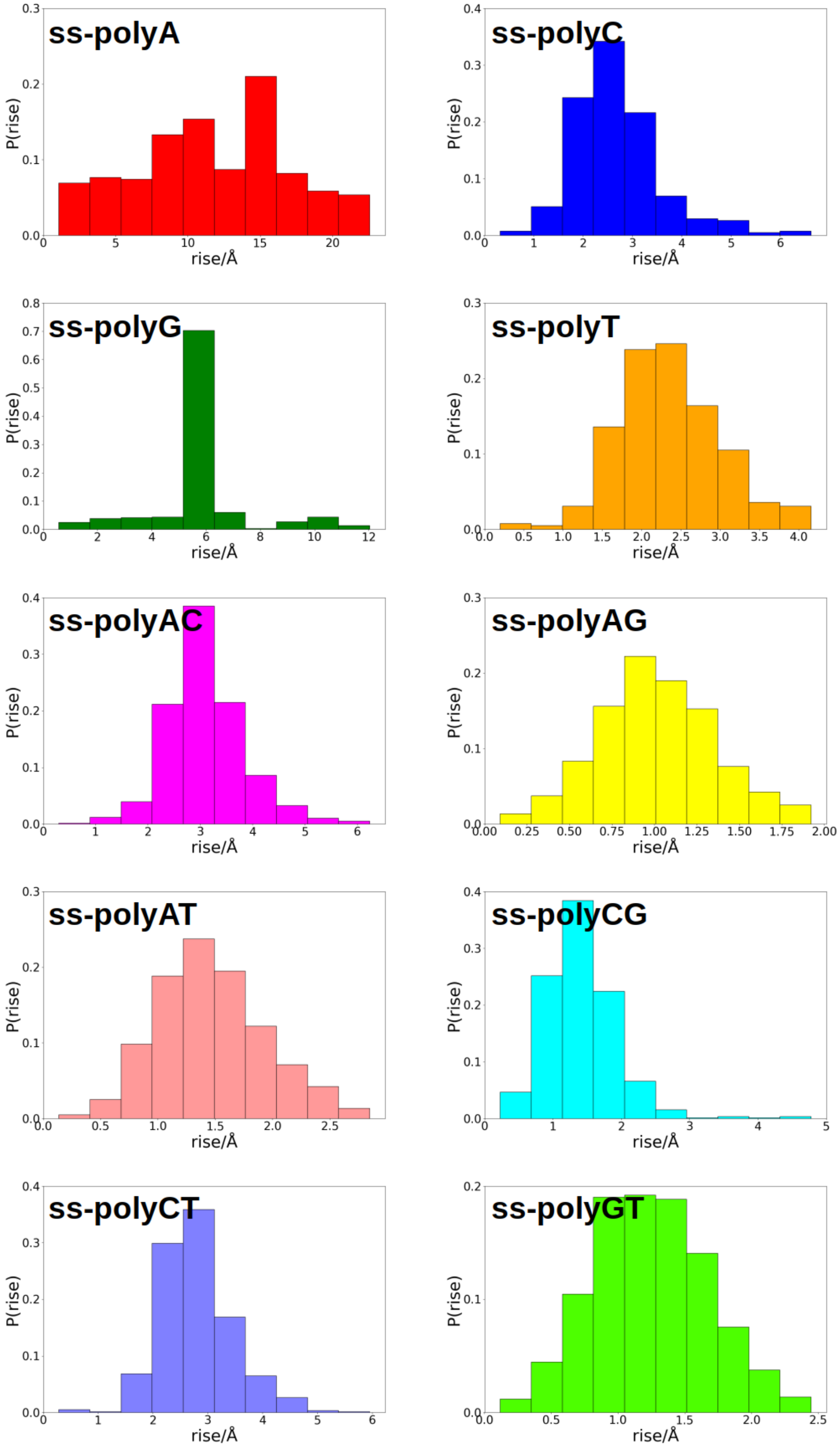

Figure S6: Distributions of the sampled rise parameter along the simulations of each ss-DNA strand.

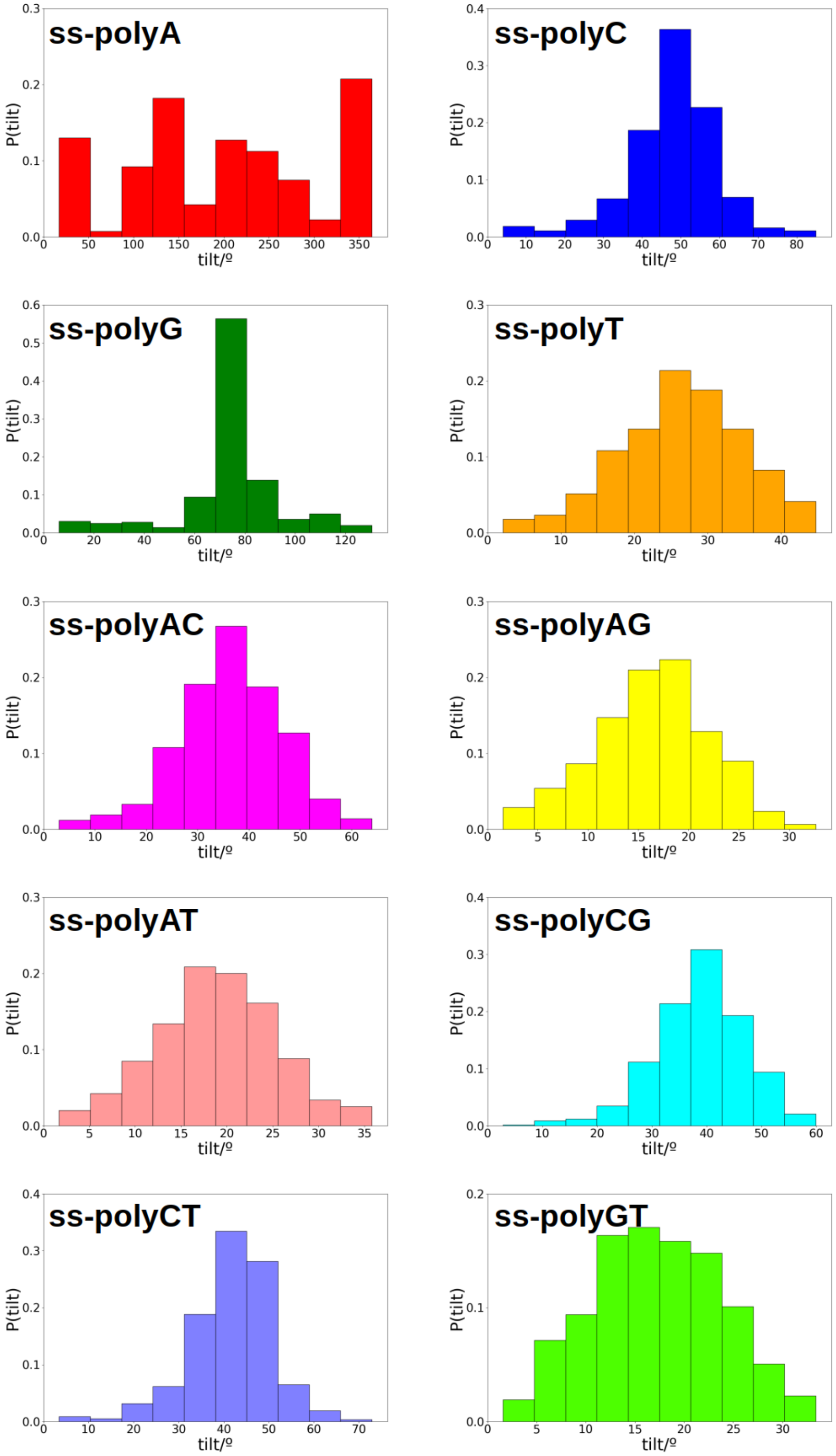

Figure S7: Distributions of the sampled tilt parameter along the simulations of each ss-DNA strand.

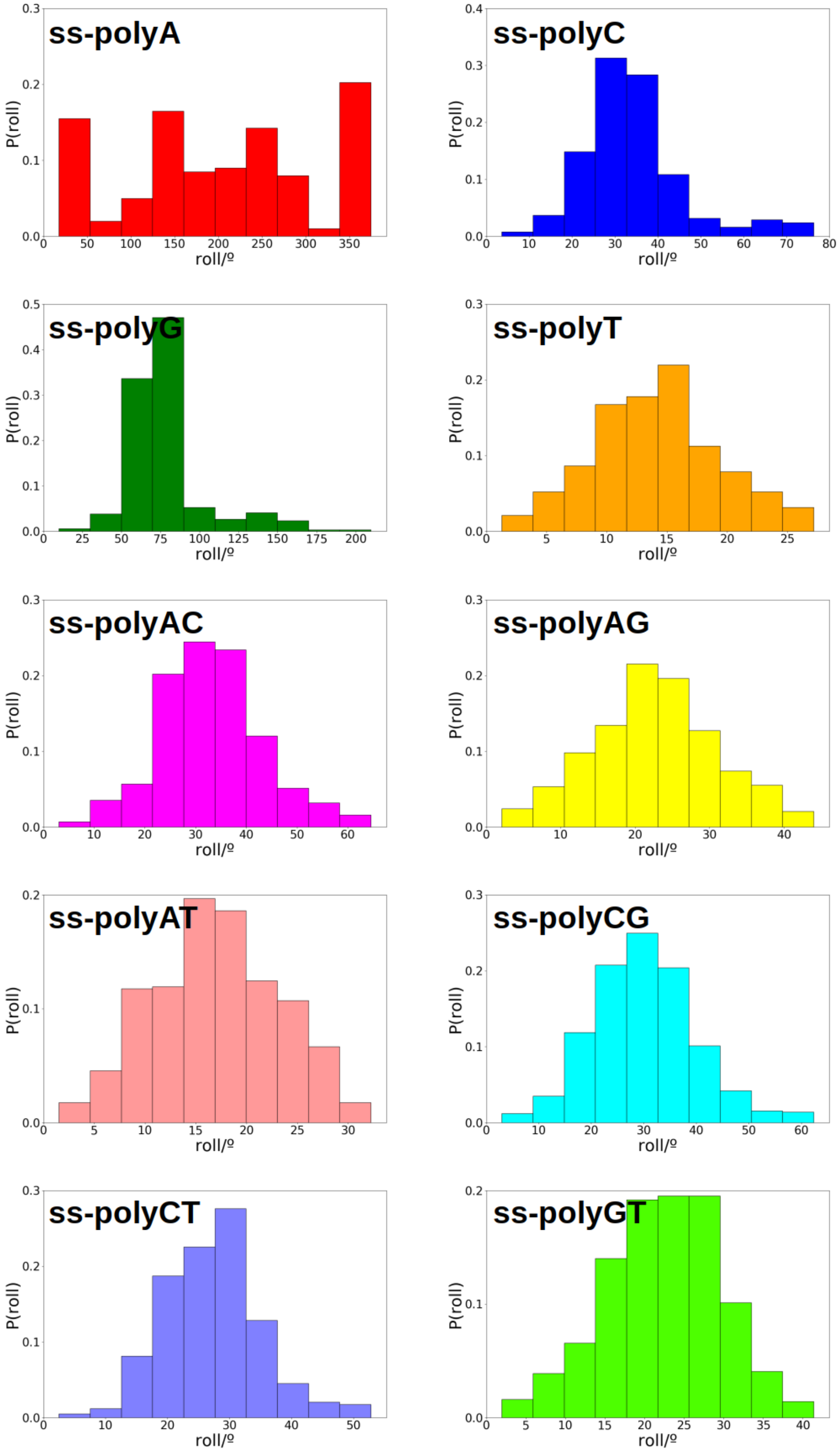

Figure S8: Distributions of the sampled roll parameter along the simulations of each ss-DNA strand.

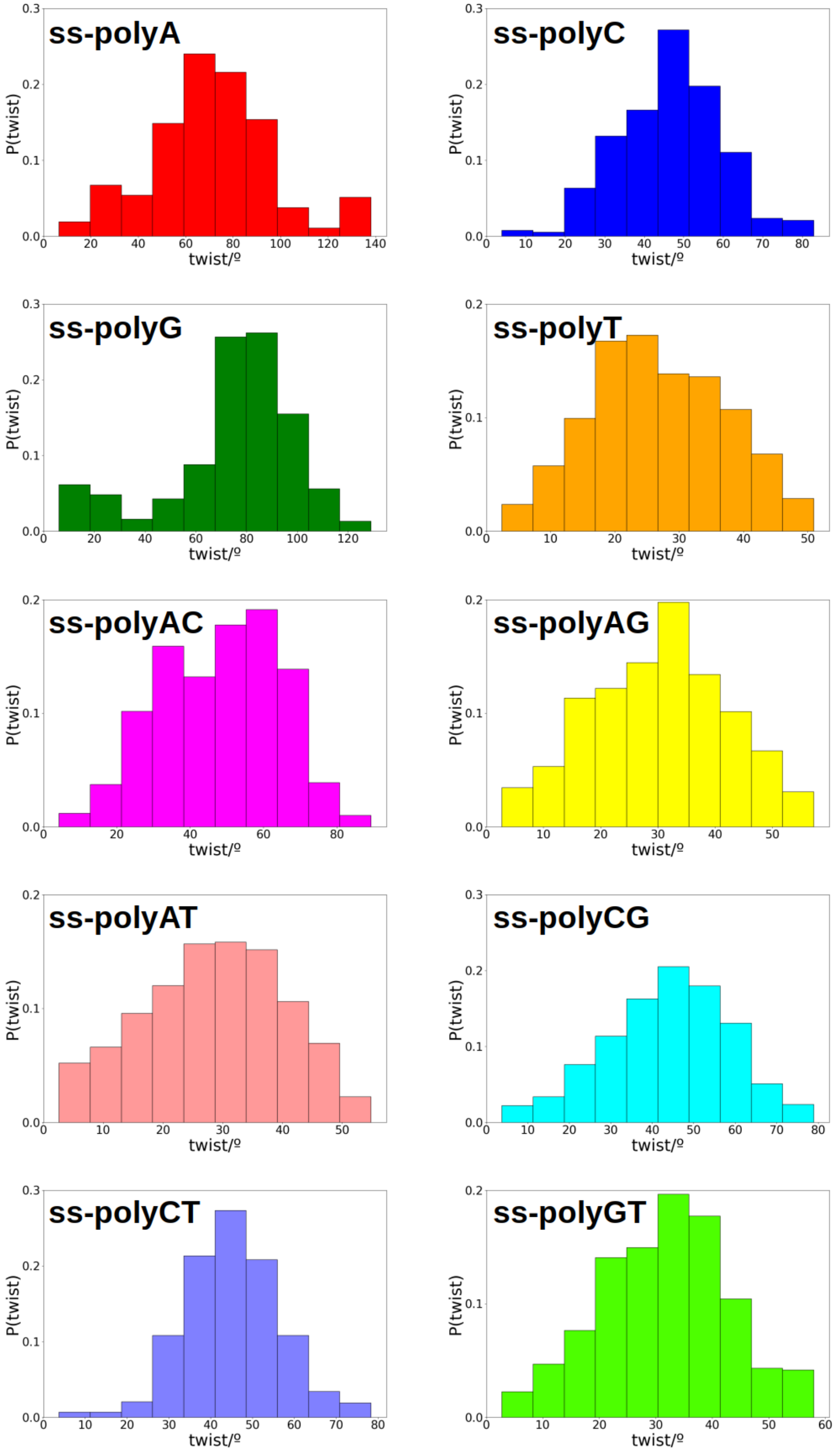

Figure S9: Distributions of the sampled twist parameter along the simulations of each ss-DNA strand.

affected because of the motion of the perpendicular nucleobase to the center of the π-system of the horizontal one. This trend can be also observed, to a lesser extent, for the simulation of ss-polyG. These conformational analysis show that other accessible conformational region has been explored for the ss-polyA and ss-polyG systems. However, in view of the results of Figure S1, the redox properties of the strands are similar independently from these two minima regions. Figures S4-S9 support this explanation. All the distributions of the sampled parameters show a gaussian shape except those associated to ss-polyA and ss-polyG. These two systems have accessed to a t-shape conformation so the distributions of values of the parameters are distorted since there exists a mixture of the normal parameters of both conformations.

Finally, an analysis was conducted in order to find correlations between each inter-base pair parameter and the intermolecular delocalization number. This analysis is shown for the case of ss-polyGT in Figure S10 as a representative example. It is possible to observe that there is a large distribution of values of delocalization number for small intervals of the parameters. This means that delocalization can be relevant or not for every explored value of each inter-base pair parameter. This fact can be associated to the coperativity of several parameters in order to allow for delocalization. In other words, for a specific value of one parameter, delocalization can be influenced by the values of the other five parameters giving rise to some situations in which delocalization is significant and some others in which is not. Consequently, no information could be extracted about the influence of each parameter to the delocalization using this procedure. However, it was possible to associate relationships between pairs of parameters in order to maximize the delocalization of the hole. This is deeply discussed in the main text.

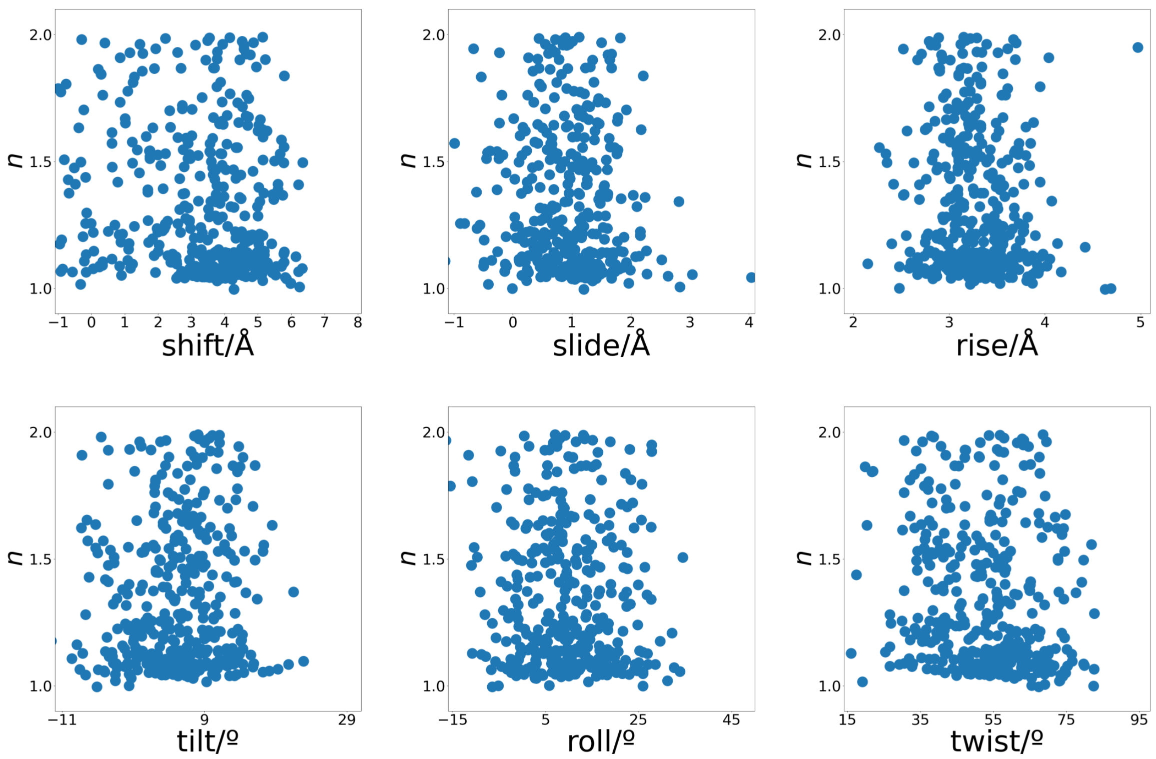

Figure S10: Graphical representation of the relative delocalization between pairs of adjacent nucleobases with respect to each inter-base pair parameters for the ss-polyGT system.

3 Relation between Structural Parameters in Absence of Delocalization

Figure S11I displays a similar representation of the existing correlations between inter-base pair parameters to the one shown in Figure 6. In this case, the representation is done for frames in which the delocalization number n is lower than 1.1. Figure S11II accounts for all the points regardless the amount of delocalization. As a result, it is possible to observe natural correlations that exist in ss-DNA. The following positive correlations can be frequetly seen among the systems: shift/twist, slide/twist and shift/slide. On the other hand, a clear negative correlation can be distinguished: rise/tilt. These correlations are the same ones discussed in the main manuscript, when only the frames with large delocalization numbers

are considered. However, on those cases of large delocalization the structural correlations are found to be more frequent, indicating that they favour the delocalization of the positive hole.

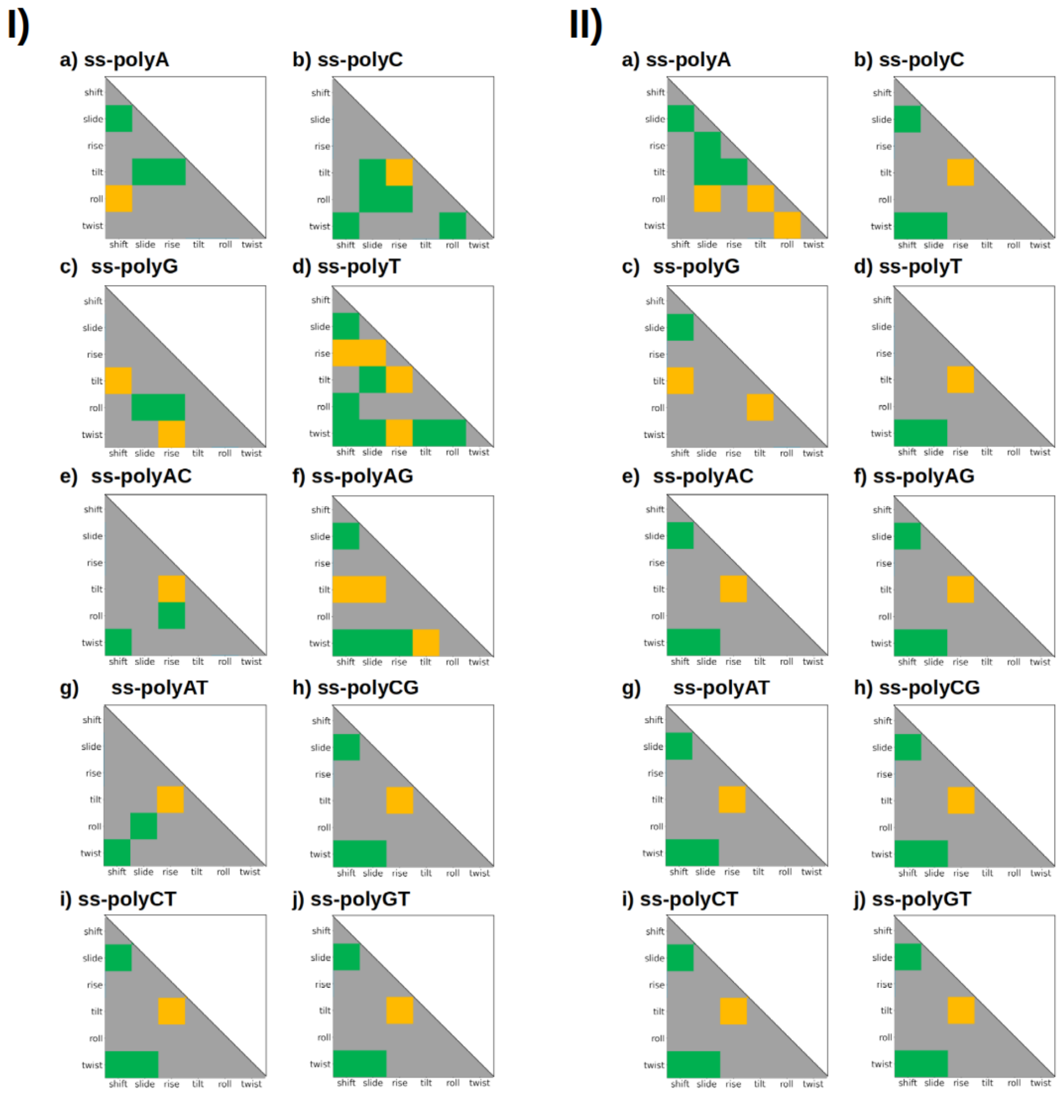

Figure S11: Coloured matrix representation of the existing correlations between inter-base pair parameters in ss-DNA I) in absence of delocalization II) regardless delocalization. Since the matrices are symmetric, only the lower triangle is displayed. Color code: green accounts for positively correlation, orange represents negatively correlations and gray refers to non existing correlation.
